# The spliceosome U2 snRNP factors promote genome stability through distinct mechanisms; transcription of repair factors and R-loop processing

**DOI:** 10.1038/oncsis.2016.70

**Published:** 2016-12-19

**Authors:** M Tanikawa, K Sanjiv, T Helleday, P Herr, O Mortusewicz

**Affiliations:** 1 Science for Life Laboratory, Division of Translational Medicine and Chemical Biology, Department of Medical Biochemistry and Biophysics, Karolinska Institutet, Stockholm, Sweden

## Abstract

Recent whole-exome sequencing of malignancies have detected recurrent somatic mutations in U2 small nuclear ribonucleoprotein complex (snRNP) components of the spliceosome. These factors have also been identified as novel players in the DNA-damage response (DDR) in several genome-wide screens and proteomic analysis. Although accumulating evidence implies that the spliceosome has an important role in genome stability and is an emerging hallmark of cancer, its precise role in DNA repair still remains elusive. Here we identify two distinct mechanisms of how spliceosome U2 snRNP factors contribute to genome stability. We show that the spliceosome maintains protein levels of essential repair factors, thus contributing to homologous recombination repair. In addition, real-time laser microirradiation analysis identified rapid recruitment of the U2 snRNP factor SNRPA1 to DNA-damage sites. Functional analysis of SNRPA1 revealed a more immediate and direct role in preventing R-loop-induced DNA damage. Our present study implies a complex interrelation between transcription, mRNA splicing and the DDR. Cells require rapid spatio-temporal coordination of these chromatin transactions to cope with various forms of genotoxic stress.

## Introduction

The splicing of pre-mRNA is a highly dynamic and flexible process carried out by large ribonucleoprotein complexes (RNPs), the ‘spliceosome'. The spliceosome is composed of uridine-rich small nuclear RNPs (snRNPs), U1, U2, U4/U6 and U5. Besides the snRNPs, the human spliceosome contains >150 different proteins. During splicing, the spliceosome is stepwise assembled from the U1/U2 snRNPs, U4/U6, the U5 tri-snRNP and the Prp19 complex on pre-mRNA consensus sequences and performs intron excisions and exon-ligations.^1, 2, 3^

Recent whole-exome sequencing studies have detected recurrent somatic mutations in components of the spliceosome in myelodysplastic syndromes,^4^ chronic lymphocytic leukemia,^5^ pancreatic cancer, breast cancer,^6^ lung adenocarcinoma,^7^ renal clear cell carcinoma^8^ and uveal melanoma.^9^ Notably, mutated components of the spliceosome were mainly detected in the U2 snRNP and U2-related proteins, which form the splicing A complex and are engaged in the initial step of splicing.^4^ This surprisingly high mutation frequency strongly suggests that the compromised function of the spliceosome is an emerging hallmark of cancer and neoplastic diseases.

Genomic instability is recognized as a characteristic of most solid tumors and adult-onset leukemia. To counteract DNA damage and maintain genome stability, cells have evolved a complex cellular DNA-damage response (DDR). Recently, a novel layer of complexity in the cellular response to DNA damage has emerged with the involvement of RNA metabolism. Several large-scale genetic and proteomic screens have revealed that RNA-binding proteins involved in different steps of mRNA life, transcription, splicing and translation, can affect genome stability. Proteomic analysis designed to identify human and mouse proteins phosphorylated by ataxia telangiectasia mutated (ATM) and ATR (ATM-Rad3 related) in response to DNA damage, detected a large number of proteins involved in RNA metabolism.^10^ More recently, another proteomic study, which quantified DNA damage-induced changes in phosphoproteome, acetylome and proteome, identified a significant fraction of the hits corresponding to proteins involved in RNA metabolism.^11^ Genome-wide small interfering RNA (siRNA)-based screens to detect novel regulators of homologous recombination (HR) also identified several components of the spliceosome among the top hits.^12,13^ Pederiva *et al.* recently identified the ubiquitin ligase RNF8 as a DNA repair factor sensitive to splicing inhibition^14^ and Adamson *et al.* reported the recruitment of RNA-binding proteins, including splicing factors, to DNA-damage sites, which suggests that these proteins might directly contribute to the DDR. However, the precise role of these splicing factors in DNA repair is still not completely understood.

Based on the results from two published genome-wide siRNA screens for HR factors,^12, 13^ we set out to further elucidate the role of splicing factors in promoting genome stability. We demonstrate that the main reason for the defects observed in the DDR upon depletion of U2-splicing factors is owing to depletion of essential repair proteins caused by downregulation of transcription. Careful examination of cellular phenotypes combined with time-resolved knockdown experiments and live-cell imaging revealed an additional, R-loop dependent, effect on genome stability. Finally, we show that the splicing factor SNRPA1 is recruited to laser-induced DNA-damage sites and prevents R-loop-induced DNA damage. We conclude that splicing factor depletion results in immediate formation of R-loops and subsequent DNA damage, which is ultimately overpowered by global attenuation of transcription and protein depletion.

## Results

### Splicing factors are among the top hits in two different genome-wide screens for HR repair factors

**Figure 1:**
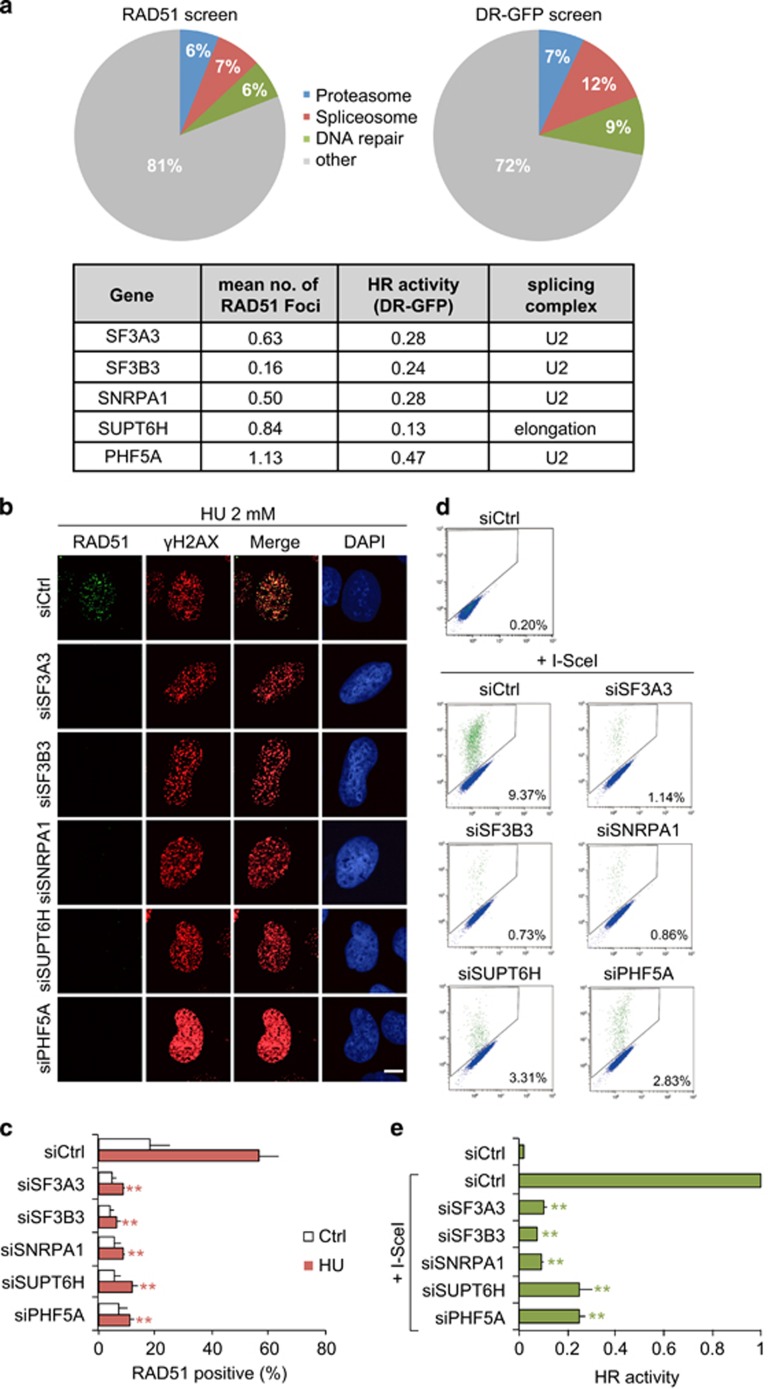
Splicing factors are among the top hits in two different genome-wide screens for homologous recombination repair factors. (**a**) The spliceosome scores high in both a RAD51 foci screen and DR-GFP screen. Pie charts for the top 100 genes from two different genome-wide screens and list of splicing factors analyzed in this study. (**b**) U2OS cells were transfected with indicated siRNA and after 48 h treated with or without 2 mM HU for 24 h. Cells were fixed and immunostained for RAD51. Splicing factor knockdown reduces RAD51 foci formation at HU-induced DNA-damage sites. (**c**) RAD51-positive cells (>12 foci) were quantified for each condition. More than 400 cells were counted. (**d** and **e**) HR reporter cells (DR-GFP_U2OS) were transfected with indicated siRNA. Forty-eight hours later, I-SceI expression plasmid was transfected and GFP-positive cells were measured after another 48 h. HR repair was significantly impaired in splicing factors depleted cells. Error bars represent s.e.m. from three independent experiments (*n*=3). Statistically significant differences between control siRNA and splicing factor siRNA-treated cells were determined using Student's *t*-test, *P<0.05, **P<0.01. Scale bar is 10 μm.

We aimed to identify novel HR repair factors using the data of two published siRNA screens.^12, 13^ Analysis of the top 100 hits of both screens revealed an enrichment of spliceosome components together with well-known DNA repair factors and proteins of the proteasome (Figure 1a). Interestingly, among the top hit candidate spliceosome genes, proteins of the U2 snRNP complex and U2-related proteins were enriched (Supplementary Figure S1a). We decided to investigate four U2 snRNP and U2-related splicing factors and one elongation factor in greater detail (Figure 1a). First we set out to validate the impaired RAD51 recruitment and HR repair (DR-GFP assay) deficiency originally used in both screens. siRNA-mediated depletion of splicing and elongation factors in U2OS cells (Supplementary Figure S1b) lead to strong impairment of RAD51 recruitment to collapsed replication forks induced by hydroxyurea (HU) (Figure 1b and c, and Supplementary Figure S1c). Splicing factor depleted cells also showed severe downregulation of HR in the DR-GFP assay (Figure 1d and e).

### Depletion of splicing factors affects DNA repair through downregulation of repair factor transcription

**Figure 2:**
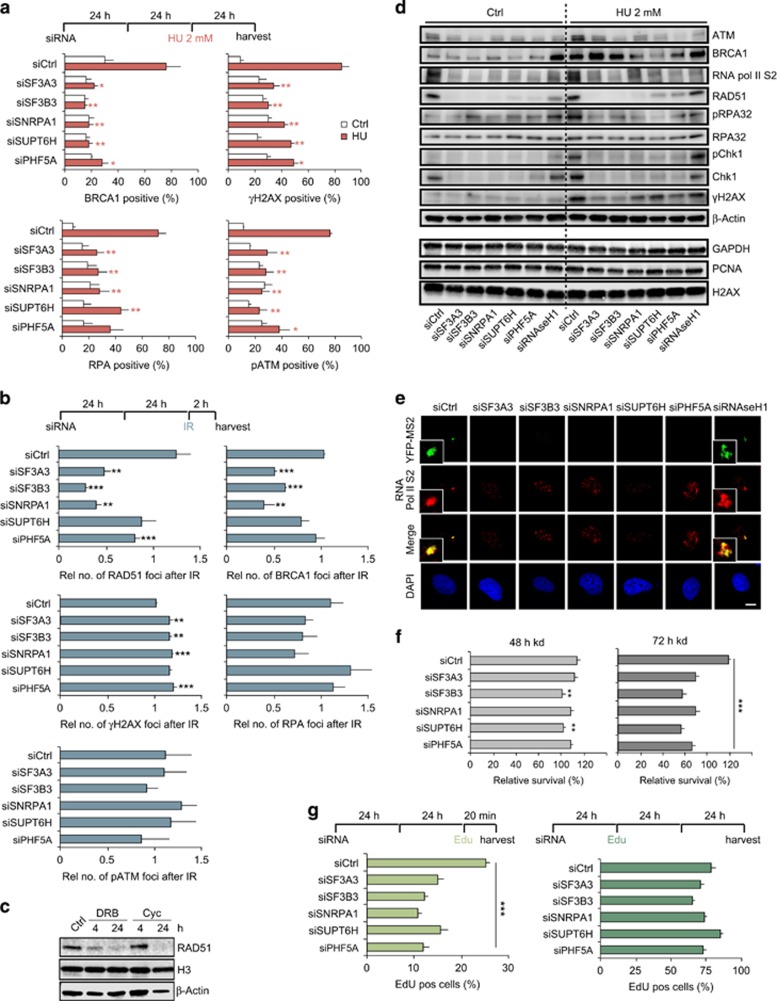
DNA repair is impaired in splicing factor depleted cells through downregulation of repair factor transcription. (**a**) U2OS cells were transfected with indicated siRNA and after 72 h fixed and immunostained with anti-γH2AX, pATM, BRCA1 and RPA antibodies. HU induced γH2AX and pATM accumulation at DNA-damage sites is reduced in splicing factor depleted cells. Quantification of γH2AX (>12 foci), pATM (>8 foci), RPA (>12 foci) and BRCA1 (>12 foci) positive cells. For each condition, >400 cells were analyzed. (**b**) Irradiation induced RAD51 and BRCA1 foci formation is significantly reduced, whereas γH2AX is increased in splicing factor depleted U2OS cells. For each condition, >3000 cells were analyzed and relative number of foci normalized to siCtrl are displayed (Rel. no.). (**c**) RAD51 protein levels are reduced after translation or transcription inhibition by Cyclohexamide or DRB. U2OS cells were treated with DRB or Cyclohexamide for either 4 or 24 h, harvested and probed for RAD51 expression levels. (**d**) U2OS cells were transfected with indicated siRNA. After 48 h cells were incubated with 2 mM HU for 24 h and then collected and analyzed by western blotting. Knockdown of splicing factors affects expression of DNA repair proteins. (**e**) 2-6-3 reporter cells, which visualize nascent transcripts, were transfected with indicated siRNA, and 48 h later treated with 1 μg/ml doxycycline. After 5 h cells were fixed for analysis of YFP-MS2 accumulation, which indicates active transcription. In splicing factor depleted 2-6-3 reporter cells, doxycycline inducible transcription was attenuated. (**f**) Cellular survival after splicing factor knockdown for 48 or 72 h is shown relative to siCtrl. (**g**) Short EdU pulse (20 min) reveals slight reduction in the number of replicating cells in splicing factor depleted cells, whereas longer EdU treatment shows completion of S-phase within 24–48 h. Error bars represent s.e.m. from three independent experiments (*n*=3). Statistically significant differences between control siRNA and splicing factor siRNA-treated cells were determined using Student's *t*-test, *P<0.05, **P<0.01, ***P<0.001.

To determine how the DDR is affected by the loss of splicing factors, we analyzed the recruitment of other DNA repair factors to HU-induced DNA-damage sites. In addition to RAD51, recruitment of BRCA1 and RPA, and phosphorylation of ATM and H2AX were also severely affected in splicing factor depleted cells (Figure 2a and Supplementary Figure S2a and b), whereas 53BP1 showed no significant change (Supplementary Figure S2c and d). Reduced recruitment of RAD51 and BRCA1 could also be observed in ionizing radiation (IR)-treated cells depleted for splicing factors, whereas γH2AX formation was increased (Figure 2b).

The deficiency in repair factor recruitment prompted us to study the effect of splicing factor depletion on the expression of DNA repair genes. We found reduced expression of DDR factors like ATM, Chk1, BRCA1 and RAD51 (Figure 2d) in both control and HU-treated cells, with RAD51 protein levels being severely depleted. Furthermore, we detected reduced phosphorylation of Serine 2 at the C-terminal domain of RNA polymerase II (Figure 2d), a marker for elongating RNA Polymerase II and active transcription. We also confirmed this attenuated transcription in splicing and elongation factor depleted cells using the 2-6-3 reporter cell line. (Figure 2e).^15^ In brief, upon activation YFP-tagged MS2 binds to the MS2 stem loop structure in the transcribed RNA and thus allows the visualization of nascent transcription in this cell line. It is interesting to note that other proteins like RPA, PCNA, glyceraldehyde 3-phosphate dehydrogenase (GAPDH) and beta-Actin, as well as Histones, showed no reduction in expression levels, indicating that the reduction in protein levels upon splicing factor depletion might be specific to (HR) repair proteins (Figure 2d). Blocking either transcription or translation using DRB or Cyclohexamide also resulted in severe reduction of RAD51 protein levels (Figure 2c). The repair defects observed in HU-treated cells and the DR-GFP assay could also result from a reduced number of replicating cells. We found a slight reduction in S-phase cells after a short 20 min EdU pulse in splicing factor depleted cells (Figure 2g). However using longer EdU incubation times (24–48 h) revealed that splicing factor depleted cells go through S-phase albeit likely at a slower rate (Figure 2g). Propidium iodide analysis by fluorescence-activated cell sorting also showed a minor reduction in S-phase cells (Supplementary Figure S3a). Furthermore we did not observe a strong effect on cell survival after splicing factor knockdown (Figure 2f). We conclude that the marked reduction in HR efficiency and repair factor recruitment after HU or IR is at least partly caused by attenuated transcription of repair factors.

### Splicing inhibition reduces HR through attenuating transcription

**Figure 3:**
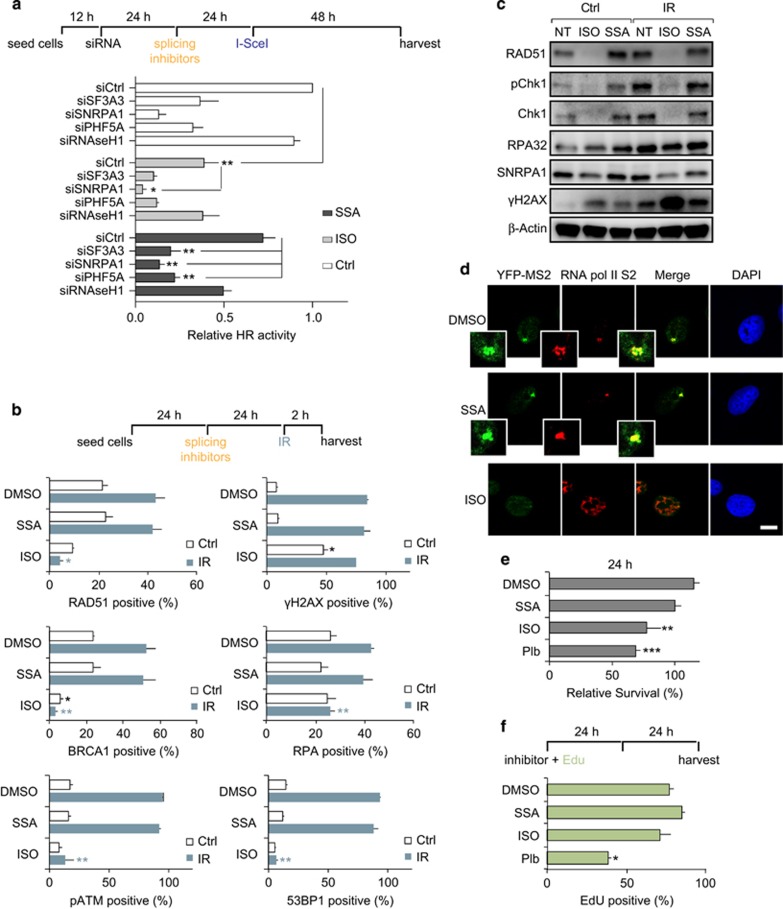
Splicing inhibitor Isoginkgetin (ISO) strongly reduces homologous recombination through attenuating transcription. (**a**) DR-GFP_U2OS cells were transfected with indicated siRNA. Twenty-four hours later, splicing inhibitors, ISO and Spliceostatin A (SSA) were added where indicated (ISO: 30 μM, SSA: 100 nM) and 24 h later, I-SceI expression plasmid was transfected. Forty-eight hours after I-SceI transfection, cells were collected for FACS analysis. Although the splicing inhibitor ISO significantly decreases HR, SSA has less-dramatic effects. Combination of splicing factor knockdown and inhibitors leads to additional HR defects. (**b**) U2OS cells were incubated with ISO or SSA and 24 h later exposed to ionizing radiation (2 Gy). Two hours later, cells were fixed and immunostained with the indicated antibodies. Quantification of γH2AX (>12 foci), pATM (>12 foci), RAD51 (>12 foci), BRCA1 (>12 foci), RPA (>12 foci) and 53BP1 (>12 foci) positive cells is shown. For each quantification, >400 cells were analyzed. (**c**) Cells were treated as in (**b**) and analyzed by western blotting. ISO treatment leads to reduced expression of DNA repair proteins as observed in cells depleted for splicing factors by siRNA. (**d**) 2-6-3 reporter cells were incubated with splicing inhibitors (ISO: 33 μM, SSA: 100 nM), and after 24 h treated with 1 μg/ml doxycycline for 5 h. Cells were fixed for analysis of YFP-MS2 accumulation. In ISO-treated cells, doxycycline induced transcription was significantly attenuated similar to splicing factor depleted cells. (**e**) Cellular survival after splicing inhibitor treatment. (**f**) EdU incorporation experiment shows completion of S-phase within 48 h after treatment with SSA and ISO, whereas Plb treatment results in decrease in S-phase cells. The error bars represent s.e.m. from two-three independent experiments. Statistically significant differences were determined using Student's *t*-test, *P<0.05, **P<0.01, ***P<0.001. Scale bar is 10 μm.

We next tested the effect of the splicing inhibitors Isoginkgetin (ISO) and Spliceostatin A (SSA) on DNA repair. The biflavonoid ISO is thought to inhibit the progression of the spliceosome complex A to B transition, but the target protein is not yet known.^16^ SSA is the methylated form of FR901464, which shows not only potent cytotoxic activity against a number of different human solid tumor cells but also prolongs the life of tumor-bearing mice.^17^ The target protein of SSA is SF3B1, which is reported to be a commonly mutated U2 snRNP component and is thought to inhibit formation of spliceosome complex A interfering with early steps of spliceosome assembly.^18,2^ Concentrations and incubation times (ISO; 33 μM, SSA; 100 nM, both for 24 h) were chosen according to previous reports.^16^ The two splicing inhibitors showed varying effects on HR and transcription. ISO strongly reduced HR in the DR-GFP assay, whereas SSA had only a slight effect (Figure 3a and Supplementary Figure S3b). ISO treatment like siRNA-mediated depletion of splicing factors lead to a strong reduction of pATM, RAD51, BRCA1 and RPA foci formation. These effects were not observed in SSA-treated cells (Figure 3b). To test whether treatment with SSA or ISO causes DNA damage by itself, we analyzed cells after 26 h of incubation. ISO treatment caused a strong increase in γH2AX and reduction of DDR protein expression similar to splicing factor depletion (Figure 3c). Pladienolide B, another splicing inhibitor, had similar effects as ISO (Supplementary Figure S3c). SSA treatment for 24 h, had no effect on DDR factors recruitment to irradiation induced DNA-damage sites at early time points (Figure 3b) but lead to induction of γH2AX, and reduction of protein expression levels after IR (Figure 3c). ISO-treated cells showed clear attenuated transcription in the 2-6-3 reporter cell line, which probably leads to downregulated expression of DDR genes (Figure 3d). ISO, as well as pladienolide B both reduced cellular survival, whereas SSA had nearly no effect (Figure 3e). Long-term EdU incorporation experiments revealed that SSA- and ISO-treated cells go through S-phase within a treatment period of 48 h, whereas S-phase cells are reduced in pladienolide B-treated cells (Figure 3f). We conclude that HR deficiency and impaired recruitment of DDR factors to sites of DNA damage is likely due to attenuated transcription. Importantly, combining ISO and knockdown of splicing factors showed additive effects on HR, which points to a direct involvement for splicing factors in HR (Figure 3a).

### Recruitment and dissociation of splicing factors at laser-induced DNA-damage sites

**Figure 4:**
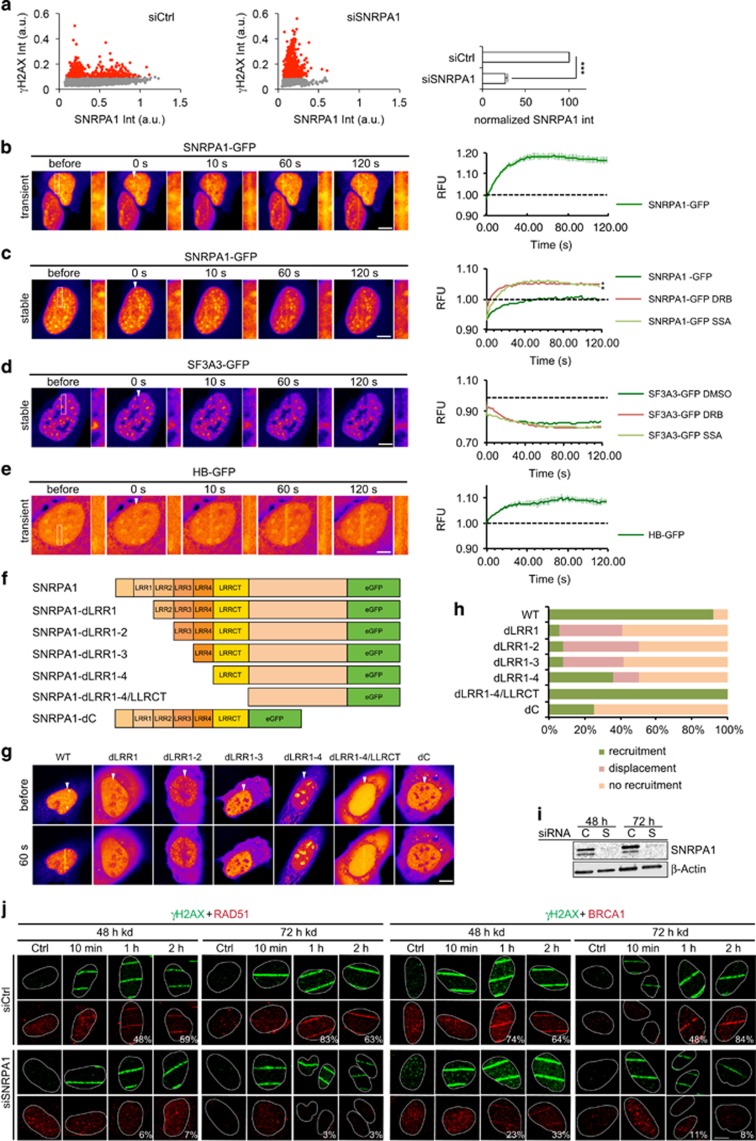
Recruitment kinetics of SNRPA1 and SF3A3 after laser microirradiation and SNRPA1-dependent recruitment of RAD51 and BRCA1 to laser tracks. (**a**) Image-based cytometry reveals increased γH2AX formation after 72 h SNRPA1 knockdown in U2OS cells. SNRPA1 knockdown effciency is shown in the bar graph. U2OS cells transiently (**b**) or stably (**c**) expressing SNRPA1-GFP were microirradiated and protein recruitment followed in real-time. For transcription and splicing inhibition cells were pretreated with DRB (50 μM) or SSA (100 nM) for at least 1 h before damage induction. Representative confocal images and recruitment kinetics are shown. (**d**) As in (**b** and **c**) but in cells stably expressing SF3A3-GFP. (**e**) Generation of R-loops at laser tracks visualized by HB-GFP recruitment. HB-GFP recruitment kinetics resemble SNRPA1-GFP. (**f**) Schematic of SNRPA1 deletion constructs. (**g**) Representative confocal images of recruitment of wild-type and mutated GFP-tagged SNRPA1 after laser microirradiation. (**h**) Quantification of recruitment, non-recruitment and displacement of GFP-tagged SNRPA1 fusions. Ten cells were analyzed per condition. (**i**) Confirmation of siRNA-mediated depletion of SNRPA1 after 48 and 72 h knockdown in cells used in (**j**). (**j**) Recruitment of RAD51 and BRCA1 in control and SNRPA1-depleted U2OS cells. U2OS cells were transfected with control or SNRPA1 siRNA and 48 or 72 h later microirradiated, fixed after indicated repair times and stained for γH2AX, RAD51 and BRCA1. Representative confocal images and percentage of cells displaying RAD51 and BRCA1 recruitment are shown. Scale bar 5 or 10 μM (**i**). Statistically significant differences were determined using Student's *t*-test, *P<0.05, **P<0.01. Error bars represent s.e.m. RFU (relative fluorescence units).

Splicing factor depletion and splicing inhibition both induce γH2AX. Using Image-based cytometry, we could show that reduced levels of SNRPA1 in particular correlate with γH2AX induction (Figure 4a). To test the direct involvement of splicing factors in the DDR we microirradiated U2OS cells transiently or stably transfected with GFP-tagged SNRPA1 or SF3A3 and analyzed their recruitment in real-time. Interestingly, we could detect recruitment of SNRPA1-GFP only in transiently transfected cells (Figure 4b), whereas recruitment was barely detectable or absent in stably transfected cells (Figure 4c). Expression levels of SNRPA1-GFP were generally ~2–4-fold higher in transiently transfected cells compared with stable cells. GFP alone was not recruited to laser-induced DNA-damage sites under the conditions used (Supplementary Figure S4d). We therefore speculated that SNRPA1-GFP accumulation at DNA repair sites can only be detected when SNRPA1 is present in excess and not engaged in other cellular processes as has been previously described for other splicing factors.^19^ To test this hypothesis we treated SNRPA1-GFP stable cell lines with transcription or splicing inhibitors and followed accumulation of SNRPA1-GFP over time. Under these conditions we could detect recruitment of SNRPA1-GFP in stably transfected cells, indicating that SNRPA1 molecules that are not engaged in transcription or splicing readily accumulate at DNA-damage sites (Figure 4c). Deletion of the N-terminal Leucine-rich repeats (LRR1-4 and LRRCT) prevented efficient relocalization of GFP-tagged SNRPA1 to laser tracks (Figure 4f and h and Supplementary Figure S4a). In fact, formation of anti-stripes could be observed in some cases, indicating dissociation from DNA-damage sites. LLRs are considered to mediate protein–protein interactions, suggesting that SNRPA1 is recruited to DNA-damage sites via these domains. However, we could also observe recruitment of the isolated C-terminal domain of SNRPA1, which lacks all four LLRs as wells as the LRRCT domain (Figure 4f and h). Taking together, our results suggest that SNRPA1 recruitment to DNA-damage sites could be mediated through at least two different mechanisms.

In contrast to SNRPA1, SF3A3-GFP dissociated from laser tracks upon microirradiation. When treated with transcription or splicing inhibitors these anti-stripes upon microirradiation became even more apparent (Figure 4d and Supplementary Figure S4b). As SNRPA1 is involved in splicing and R-loops may occur as products of unsuccessful splicing, we hypothesized that SNRPA1 might specifically be recruited to R-loops generated at DNA-damage sites. To test this we stably expressed HB-GFP in U2OS cells, which is a fusion of the DNA–RNA hybrid-binding (HB) domain of RNaseH1 and enhanced green fluorescent protein and thus can be used to label R-loops in living cells.^20^ Indeed we found that the recruitment kinetics of SNRPA1 resemble the recruitment kinetics of HB-GFP (Figure 4e and Supplementary Figure S4c), indicating that binding of R-loops by SNRPA1 might be one of the recruitment mechanisms. Taken together, we unraveled two very distinct responses of splicing factors to local DNA-damage induction. Although SNRPA1 is recruited to laser tracks and is thus likely involved in the DDR, potentially mediating repair at R-loops, SF3A3 dissociates from DNA-damage sites, indicating disassembly of the splicing machinery to allow access of repair proteins.

### SNRPA1 knockdown impairs recruitment of BRCA1 and RAD51 to DNA-damage sites

**Figure 5:**
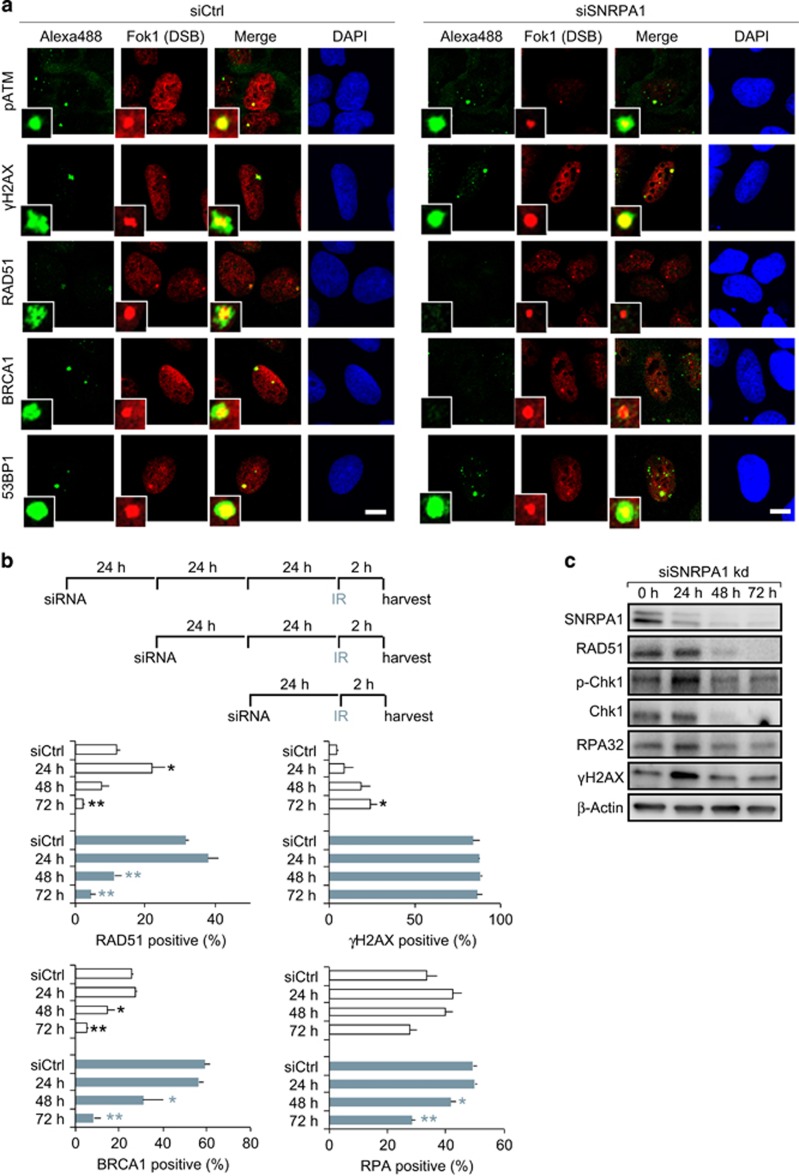
SNRPA1 knockdown impairs BRCA1 and RAD51 accumulation at endonuclease (FokI) cleaved single DSB sites and radiation-induced DSB sites. (**a**) Visualization of endonuclease-mediated DSB induced by Shield 1 addition in 2-6-5 reporter cells with mCherry-LacI-FokI fusion protein expression. Cells were transfected with indicated siRNA, and 48 h later 4-OHT and Shield 1 were added for 5 h to induce mCherry-LacI-FokI expression. Cells were fixed and immunostained with indicated antibodies. In SNRPA1-depleted cells, BRCA1 and RAD51 accumulation to endonuclease cleaved single DSB sites were significantly reduced. (**b**) U2OS cells were transfected with control or SNRPA1 siRNA and after indicated time periods irradiated with 2 Gy. Two hours after irradiation, cells were fixed and immunostained. Quantification of RAD51 (>12 foci), γH2AX (>12 foci), BRCA1 (>12 foci) and RPA (>12 foci) positive cells. For each condition, more than 400 cells were analyzed. (**c**) After indicated time periods of SNRPA1 knockdown, cells were collected and analyzed by western blotting. Forty-eight hours after SNRPA1 depletion, protein expression levels of RAD51 and Chk1 were already reduced. At 24 h after siSNRPA1 transfection, γH2AX levels transiently increased and then declined. The error bars represent s.e.m. from three independent experiments (*n*=3). Statistically significant differences between cells treated with control or splicing factor siRNA were determined using Student's *t*-test, *P<0.05, **P<0.01. Scale bar is 10 μm.

We further investigated a direct involvement of SNRPA1 in DNA repair by analyzing recruitment of DNA repair factors to damage sites induced by microirradiation, endonuclease induction, irradiation and HU-treatment in SNRPA1-depleted cells. Neither RAD51 nor BRCA1 could be detected at laser tracks in SNRPA1-depleted cells (48 and 72 h knockdown), whereas a clear recruitment was observed in control cells (Figure 4i and j). We next used the 2-6-5 reporter cell line^21^ to directly visualize protein recruitment to Fok1 endonuclease induced double-strand breaks (DSB). Foci formation of HR repair factors BRCA1 and RAD51 was markedly reduced in SNRPA1-depleted cells at both endonuclease and irradiation induced DSB sites. Interestingly, pATM and γH2AX foci formation, upstream of BRCA1 and Rad51, as well as 53BP1 were preserved (Figure 5a and b). HU-treated cells depleted of SNRPA1 showed a similar reduced recruitment of RAD51 and BRCA1 and diminished phosphorylation of ATM and H2AX (Supplementary Figure S5). Taken together, these data indicate that attenuated transcription may mask a direct repair defect in SNRPA1-depleted cells. To carefully analyze this potential direct role of SNRPA1 in DNA repair we followed DNA repair factor recruitment and modifications after increasing knockdown times (24, 48 and 72 h) in untreated and irradiated cells. In untreated cells, γH2AX foci were continuously increasing after prolonged time of SNRPA1 depletion indicating accumulation of DNA damage (Figure 5b), likely induced by R-loop formation (see below). We also observed a transient increase in RAD51 and RPA foci formation at 24 h SNRPA1 knockdown. This suggests the existence of exposed single stranded DNA which could be caused by R-loops. Importantly, SNRPA1 is already depleted after 24 h knockdown, whereas RAD51 protein levels are still stable and γH2AX and pChk1 are induced (Figure 2d and Figure 5c). At later time points however, RAD51 and BRCA1 foci, as well as RAD51 protein levels significantly decreased even in irradiated cells, which is likely owing to the previously observed effect on transcription (Figure 5b and c). However, as BRCA1 levels are only mildly affected after siSNRPA1 knockdown (Figure 2d), lack of BRCA1 recruitment to laser stripes (Figure 4i), FokI-induced DSB (Figure 5a) and IR-induced lesions (Figure 5b), points to a more direct role of SNRPA1 in promoting BRCA recruitment to repair sites. In summary, by performing a time course of SNRPA1 depletion we could show that besides the late occurring indirect effect on transcription of repair factors, SNRPA1 also has a more direct role in genome stability, which is apparent at early time points of SNRPA1 depletion.

### DNA damage caused by SNRPA1 depletion and SSA treatment is mediated by R-loop formation

**Figure 6:**
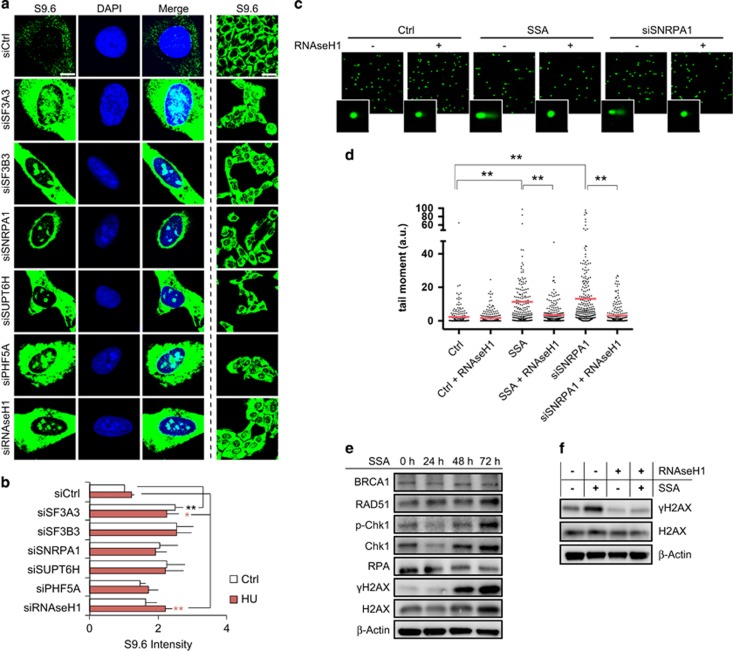
DNA-damage induction in SNRPA1-depleted and SSA-treated cells is R-loop dependent. (**a**) Splicing factor depleted U2OS cells were fixed and probed for R-loop (DNA–RNA hybrids) formation using the S9.6 antibody after 72 h of knockdown. RNAseH1-depleted cells served as positive control. (**b**) Quantification of mean S9.6 intensity per nucleus. More than 400 cells were analyzed. R-loop formation was significantly increased in splicing factor depleted cells. (**c**) Representative images of comet assays performed under alkaline conditions in U2OS cells incubated with SSA or DMSO and treated with siCtrl or siSNRPA1 in the absence (+GFP empty) and presence (+GFP-RNAseH1) of ectopic RNAseH1 expression. (**d**) Quantitative analysis of comet tail length for each condition. (**e**) U2OS cells were incubated with Spliceostatin A (SSA) for the indicated time periods and analyzed by Western blotting. Expression of most DNA repair factors was retained, whereas γH2AX was induced after 48 h incubation with SSA, indicating induction of DNA damage. (**f**) γH2AX induction in U2OS cells treated with SSA can be rescued by ectopic RNAseH1 expression. The error bars represent s.e.m. from three independent experiments (*n*=3). Statistically significant differences were determined using Student's *t*-test, *P<0.05, **P<0.01. Scale bar is 10 μm. As for comet assay, Tail moment was calculated as per cent DNA in the tail multiplied by the tail length using CometScore software (*n*=200). Data were presented using GraphPad Prism6.

In previous studies, deficiency in RNA splicing was shown to cause an increased formation of R-loops, subsequent DNA breaks and p53 activation.^22^ To determine whether depletion of U2 snRNP splicing and elongation factors results in increased formation of R-loops, we used the monoclonal antibody S9.6, which detects RNA-DNA hybrids.^23^ siRNA-mediated depletion of splicing factors induced an increase in R-loop formation in U2OS cells, which was even higher than in HU-treated cells (Figure 6a and b). R-loops can be an obstacle for strand invasion and thereby interfere with HR-mediated DNA repair.

We next analyzed DNA damage induced by splicing inhibitor treatment and SNRPA1 depletion in more detail. We decided to use the milder splicing inhibitor SSA instead of ISO for the remaining experiments, owing to the very strong effect of ISO on transcription and DNA damage induction after short incubation times (24 h) (Figure 3c and d). We could detect a clear induction of γH2AX after 48 h SSA treatment (Figure 6e). In addition, a significant increase in comet tail moments could be observed in both SSA-treated and SNRPA1-depleted cells. Interestingly, this could be completely reversed by transient RNAseH1 overexpression (Figure 6c and d). In addition, γH2AX induction detected after SSA treatment was rescued by RNAseH1 overexpression (Figure 6f). These results suggest that inhibition of the spliceosome U2 complex by SNRPA1 depletion and SSA leads to R-loop-induced DNA damage.

## Discussion

Proteins of the spliceosome have been recently identified as top hits in various DNA repair screens, but their function in the DDR still remains largely elusive. mRNA maturation, including 5′ and 3′ ends processing, splicing and transport are tightly connected with transcription. Factors involved in these processes are recruited to pre-mRNA and contribute to the mRNA processing co-transcriptionally through interaction with the C-terminal domain of RNA Polymerase II. Splicing of mRNA is subject to spatio-temporal control by transcribing polymerases, chromatin remodelers and histone marks.^24^ Therefore, the role of splicing factors in genome integrity is likely tightly connected with transcription. Here, we present two distinct mechanisms of how spliceosome U2 snRNP factors promote genome stability. First, the spliceosome maintains transcription of essential repair factors and we speculate that this is one of the main reasons why splicing factors have been enriched as positive hits in genome instability screens. The other, more immediate and direct role of the spliceosome, exemplified here by SNRPA1, is to prevent the formation of R-loop structures at sites of ongoing transcription and directly promote DNA repair.

Our study shows that U2 snRNPs, which were identified as novel HR factors, are essential for the maintenance of transcription. Depletion of a single U2 snRNP splicing factor lead to attenuated transcription and reduced expression of DDR factors like RAD51, ATM and Chk1. Splicing factor depletion furthermore resulted in severe defects in recruitment of the HR factors BRCA1 and RAD51 and HR deficiency. Interestingly, BRCA1 expression levels were not or only mildly affected by depletion of most splicing factors, whereas its recruitment to DNA-damage sites was dependent on these factors indicating a more direct role in BRCA1 recruitment.

A connection between splicing factors and HR has been suggested by proteomic analyses and genome-wide siRNA-based screens that aimed to detect novel regulators of the DDR and HR factors.^10, 11, 12, 13, 25^ RBMX, a hnRNP that associates with the spliceosome and influences alternative splicing, was identified in one of these screens^12^ and showed PARP-1 dependent transient recruitment to DNA-damage sites. In our study, among the U2 snRNP components that we identified as novel HR candidates, only SNRPA1 was recruited to laser-induced DNA-damage sites, whereas other factors like SF3A3 dissociated from laser tracks. Similar to RBMX, SNRPA1 recruitment was transient and occurred very early. Depletion of other U2 snRNP splicing components also showed strong HR impairment. This HR deficiency was likely due to attenuated transcription of RAD51. The cell cycle regulated expression of RAD51 and Chk1 may explain their sensitivity to transcription attenuation and we could show that RAD51 protein levels are unstable. Furthermore, the direct effect of splicing factor depletion most likely affects DNA replication during S-phase as bulky DNA:RNA hybrid structures would affect faithful replication of the DNA. Recruitment of HR factors to DNA damage induced by endonuclease or irradiation was also impaired. Accumulating evidence suggests that transcriptionally active chromatin more efficiently recruits HR factors through histone modifications like H3K36me3. Thus, in splicing factor depleted cells, attenuated global transcription in addition might lead to HR deficiency through changes in histone modifications like H3K36me3.^26^

Several other RNA metabolism related factors like THRAP, PPM1G and PRP19 have been identified as DDR factors by proteomic analysis,^11^ but their precise role in DNA repair remain ambiguous, with some of them being recruited and others excluded from DNA-damage sites, similar to what we observed here for SNRPA1 and SF3A3. SNRPA1 recruitment kinetics to laser-induced DNA-damage sites resembled that of HB-GFP, strongly suggesting that SNRPA1 is directly involved in R-loop-mediated DDR.

Although R-loops are important for transcriptional regulation at CpG islands and G-rich transcription pause sites,^27, 28^ they also can exert potentially harmful effects on genome integrity owing to the fragility of the displaced DNA leading strand. The processing of mRNA was reported to be one of the factors to prevent R-loop formation.^29, 30^ R-loops can lead to collisions between the transcription and replication machinery resulting in fork collapse^31, 32^ and can be processed into DSBs by XPF and XPG, two flap endonucleases involved in transcription coupled nucleotide excision repair.^33^ However, it is still unclear how the transcription coupled nucleotide excision repair recognizes and processes R-loops into DSBs. In our study treatment with SSA, an inhibitor of SF3B1, and SNRPA1 depletion both induce DNA damage, detected by alkaline comet assay. This DNA damage induction can be reversed by transient expression of RNAseH1 and strongly suggests that the U2 snRNP complex may protect the genome from R-loop-induced DNA damage. Interestingly, treatment with SSA induces γH2AX, which also can be rescued by transient expression of RNAseH1. Besides its deleterious effects on genome stability it has been recently reported that the dissociation of the core spliceosome, which we also observed in our study, and subsequent R-loop formation at DNA-damage sites are necessary steps for activating ATM signaling.^34^

In the present study, we used splicing inhibitors, time-resolved knockdown experiments and live-cell imaging to identify two main mechanisms of how U2 snRNP- and U2-related splicing proteins regulate genome integrity. The first is through enabling transcription of essential HR repair factors, whereas the second is direct contribution to the R-loop-related DDR. We also provide evidence that DNA damage caused by U2 snRNP complex inhibition could be a combination of R-loop-mediated DNA lesions and DSBs induced by an impaired DDR through attenuated transcription. This might explain how somatic mutations in the U2 snRNP splicing complex could cause cancer. R-loop-mediated DNA damage and mutations can be amplified by impaired HR repair enabling cells to deal with a defunct DDR resulting in carcinogenic transformation. Overexpression of mutant splicing factors induces abnormal mRNA splicing, leading to the generation of unspliced RNA species and induce the non-sense-mediated mRNA decay pathway. However, these mutations also lead to reduced cell proliferation with marked increase in the G2/M fraction together with enhanced apoptosis, which suggests induction of genome instability.^4^

We show here that the role of splicing factors in genome stability is tightly connected with transcription and R-loop formation. Future studies will unravel the mechanism of how R-loop-induced DNA damage on one hand, and the R-loop-mediated DDR on the other, are regulated and connected by the splicing machinery.

## Material and methods

### Cell culture, transfection and treatment

U2OS osteosarcoma cells were cultured in Dulbecco's Modified Eagle Medium (Invitrogen, Paisley, UK) supplemented with 10% fetal bovine serum and 1% antibiotics (penicillin/streptmycin), at 37 °C and 5% CO_2_ atmosphere. DR-GFP U2OS cells were cultured in Dulbecco's Modified Eagle Medium with 10% fetal bovine serum, antibiotics and 1 μg/ml Puromycin (Invitrogen). 2-6-3^15^ and 2-6-5^21^ U2OS reporter cell lines were kindly provided by Roger A. Greenberg and cultured in Dulbecco's Modified Eagle Medium with 10% Tet-System approved fetal bovine serum (Takara Bio USA, Inc., Mountain View, CA, USA). For the 2-6-3 cell line, 2 μg/ml puromycin, 100 μg/ml hygromycin and 200 μg/ml G418 were used as antibiotics while for the 2-6-5 cell line 2 μg/ml puromycin was added to the media. For siRNA-mediated knockdown, cells were transfected with 5 nM predesigned Silence Select siRNAs (Thermo Scientific, Waltham, MA, USA) or control siRNA (Qiagen, Valencia, CA, USA) using INTERFERin (Polyplus Transfection, Illkirch, France). For plasmid transfection, jetPEI (Polyplus Transfection) was used. DNA damage was induced by HU (Sigma-Aldrich, Schnelldorf, Germany), dissolved in H_2_O. Splicing inhibitors used are ISO (Millipore, Schnelldorf, Germany), SSA (AdooQ Bioscience, Irvine, CA, USA) and pladienolide B (sc391691, Santa Cruz Biotechnology, Dallas, TX, USA) dissolved in dimethyl sulfoxide (DMSO). For Tet-system, doxycycline hyclate (Dox; Sigma-Aldrich) was dissolved in H_2_O. To induce genotoxic stress, cells were treated with 2 mM HU for 24 h or exposed to 2 Gy irradiation. To inhibit the splicing machinery, cells were treated with ISO (33 μM), SSA (100 nM) or pladienolide B (100 nM, 1 μM) for indicated time periods. To visualize transcription, 2-6-3 reporter cells were treated with 1 μg/ml Dox for 5 h. Controls were treated with H_2_O or DMSO. To visualize endonuclease cleaved DSB, 2-6-5 reporter cells were used. In 2-6-5 reporters cells, FokI-mCherry-LacI was induced by 5 h incubation of Shield 1 (Clontech) and 4-OHT (Sigma-Aldrich) (both, final concentration: 1 μg/ml). All cell lines were routinely checked to be mycoplasma free.

### siRNA

siRNA-mediated knockdown was achieved using INTERFERin (Polyplus Transfect) following the manufacturers' instruction. Predesigned control siRNA (Qiagen) and Silence Select siRNAs (Thermo Scientific) were used. Each splicing factor and RNAseH1 siRNA is a pool of three target specific siRNAs.

Non-targeting: All-Stars negative control siRNA (Qiagen)

SF3A3:

#s21534 (5′-CAACUACAACUGUGAGAUUtt-3′, 3′-AAUCUCACAGUUGUAGUUGat-5′)

#s21535 (5′-CGACAUCUCACUCAUGAAAtt-3′, 3′-UUUCAUGAGUGAGAUGUCGct-5′)

#s21536 (5′-GCCCAGAGACUAUUCAGUAtt-3′, 3′-UACUGAAUAGUCUCUGGGCtc-5′)

SF3B3:

#s23847 (5′-CGUCUAUACUUACAAGCUUtt-3′, 3′-AAGCUUGUAAGUAUAGACGaa-5′)

#s23848 (5′-GUUUCAUCUGGGUUCGCUAtt-3′, 3′-UAGCGAACCCAGAUGAAACtt-3′)

#s23849 (5′-CAACCUUAUUAUCAUUGAAtt-3′, 3′-UUCAAUGAUAAUAAGGUUGtt-5′)

SNRPA1:

#s13216 (5′-CAACAGAAUAUGCCGUAUAtt-3′, 3′-UAUACGGCAUAUUCUGUUGtt-5′)

#s13218 (5′-GGUGCUACGUUAGACCAGUtt-3′, 3′-ACUGGUCUAACGUAGCACCta-5′)

#s57402 (5′-GAAGCAUUACAGAUUGUAUtt-3′, 3′-AUACAAUCUGUAAUGCUUCtt-3′)

SUPT6H:

#s13634 (5′-GGAUAGAAUAUGUAACGGUtt-3′, 3′-ACCGUUACAUAUUCUAUCCtg-5′)

#s13635 (5′-GAGCUGAGCUGUCGAUAUAtt-3′, 3′-UAUAUCGACAGCUCAGCUCtg-5′)

#s13636 (5′-GCCUAUUCCUUCAAGUAUUtt-3′, 3′-AAUACUUGAAGGAAUAGGCat-5′)

PHF5A:

#s39505 (5′-GCCUAUUAUUGUAAGGAGUtt-3′, 3′-ACUCCUUACAAUAAUAGGCat-5′)

#s39506 (5′-UGUGAUUUGUGACUCCUAUtt-3′, 3′-AUAGGAGUCACAAAUCACAca-5′)

#s39507 (5′-AGACAGACCUCUUCUAUGAtt-3′, 3′-UCAUAGAAGAGGUCUGUCUta-5′)

RNAseH1:

#s48356 (5′-CGGGAUUUAUAGGCAAUGAtt-3′, 3′-UCAUUGCCUAUAAAUCCCGaa-5′)

#s48357 (5′-CAGACAGUAUGUUUACGAUtt-3′, 3′-AUCGUAAACAUACUGUCUGta-5′)

#s48358 (5′-GGGAAAGAGGUGAUCAACAtt-3′, 3′-UGUUGAUCACCUCUUUCCCtg-5′)

siRNAs were transfected by INTERFERin (Polyplus Transfection) at a final concentration of 5 nM in U2OS, DR-GFP and 2-6-3 reporter cells.

### Antibodies

The primary antibodies used were: γH2AX (05-636, Millipore) at 1:1000 for western blotting (WB) and 1:1000 for immunofluorescence (IF), RAD51 (ABE257, Millipore) at 1:1000 for WB and 1:500 for IF, RAD51 (sc-8349, Santa Cruz Biotechnology) at 1:1000 for WB and 1:500 for IF, ATM (ab17995, Abcam, Dallas, TX, USA) at 1:1000 for WB, pATM (sc-47739, Santa Cruz Biotechnology) at 1:1000 for IF, BRCA1 (sc-642, Santa Cruz Biotechnology) at 1:1000 for WB and 1:500 for IF, 53BP1 (ab36823, Abcam) at 1:1000 for IF, S9.6 (purified from the S9.6 hybridoma cell line; gift from K Cimprich) at 1:5000 for IF, phospho Ser2 RNAPII CTD repeat YSPTSPS (ab5095, Abcam) at 1:1000 for IF, RPA32 (#2208, Cell Signaling, Waltham, MA, USA) at 1:1000 for WB and 1:1000 for IF, RPA2 (pSer33) (NB100-544, Novus, Littleton, CO, USA) at 1: 1000 for WB, pChk1 (Ser345) (#2348, Cell Signaling) at 1:1000 for WB, Chk1(2G1D5) (#2360, Cell Signaling) at 1:1000 for WB, Actin(beta) (ab6276, Abcam) at 1:5000 for WB, PCNA (sc-25280, Santa Cruz Biotechnology) at 1:1000 for WB, GAPDH (sc-25778, Santa Cruz Biotechnology) 1:2000 for WB, H2AX (ab11175, Abcam), 1:1000 for WB and SNRPA1 (NBP2-33447, Novus) at 1:250 for WB.

The secondary antibodies used were: Goat Anti-Rabbit IgG-HRP (sc2030, Santa Cruz Biotechnology) at 1:10000 for WB, sheep Anti-Mouse IgG-HRP(NA931V, GE Healthcare, Lafayette, CO, USA) at 1:10000 for WB, Goat Anti-Mouse IgG IRDye 680LT (926-68020, Li-Cor, Lincoln, NE, USA) at 1:10000 for WB and Goat Anti-Rabbit IgG IRDye 800CW (926-32211, Li-Cor) at 1:10000 for WB. For IF, goat anti-mouse IgG Alexa Flour 488 (A11001, Invitrogen), donkey anti-mouse IgG Alexa Flour 555 (A31570, Invitrogen), goat anti-rabbit IgG Alexa Flour 488 (A11008, Invitrogen), donkey anti-rabbit IgG Alexa Flour 555 (A31572, Invitrogen), goat anti-rat IgG Alexa Flour 555 (A21434, Invitrogen) at 1:1000, goat anti-rat IgG Alexa Flour 488 (A11006, Invitrogen) at 1:1000, and goat anti-rat IgG Alexa Flour 568 (A110077, Invitrogen).

### Primer sequences for real-time quantitative PCR

SF3A3:

TTTGTGGAAACTACACCTACCG (forward), GGATGCCCAAACACCTCAT (reverse)

SF3B3:

CCAGATATCCGCTGTCCAAT (forward), TCCTCTTTCAGGGTCATCCA (reverse)

SNRPA1:

TCCGCAAGTCAGAGTACTGG (forward), CCGTTTGCCCTTGAACATT (reverse)

SUPT6H:

GCTTCCTCAAGATCGACACG (forward)

ACGGGAACCATCAAGGACT (reverse)

PHF5A:

CCATCCAGGAGAAGGACAGA (forward)

CACCTCTTCTTGAAGCCGTATT (reverse)

RNAseH1:

GGATGTTCTATGCCGTGAGG (forward) TCCACCTGTGCTCTGCACT (reverse)

GAPDH:

AAGGTCGGAGTCAACGGATT (forward) CTCCTGGAAGATGGTGATGG (reverse)

### Plasmids

The GFP-RNAseH1 and HB-GFP plasmid were kindly provided by Robert J Crouch^35^ and Andrés Aguilera,^20^ respectively. We used the GFP-RNAseH1 plasmid as template to generate an untagged nuclear RNAseH1 construct. The N-terminal 27 amino acids were removed according to Cerritelli *et al.*^35^ and the PCR product was cloned with *Kpn*I/*Xho*I restriction enzymes into pCDNA3.1. C-terminal eGFP-tagged SF3A3, SNRPA1 and PHF5A plasmids were generated after PCR amplification without a Stop codon from complementary DNA derived from U2OS cells and cloned into the *Kpn*I/*Xho*I restriction sites of pCDNA3.1+. The eGFP gene was cloned in frame into the *Xho*I/*Xba*I restriction sites of pCDNA3.1+. SNRPA1 deletion constructs where cloned into the *Kpn*I/*Xho*I restriction sites of pCDNA3.1 in frame with a C-terminal eGFP tag. Gene fragments where PCR amplified from the full-length SNRPA1 expression construct. Deletions are d1 (AA1-41), d2 (AA1-64), d3 (AA1-86), d4 (AA1-110), d5 (AA1-161) and d6 (AA 162-255). Correct expression was tested by WB.

### IF

Cells plated on coverslips were fixed for 10 min at room temperature with fixative (3% paraformaldehyde, 0.1% TritonX-100, 1 × PBS). When staining for RPA, pre-extraction was performed for 5 min in ice-cold 0.5% TritonX-100 in CSK buffer (100 mM NaCl, 300 mM sucrose, 3 mM MgCl_2_, 10 mM PIPES, 1 mM ethylene glycol-bis(β-aminoethyl ether)-N,N,N′,N′-tetraacetic acid). After fixation, cells were rinsed briefly in 0.05% Tween20 in PBS twice, and then permeabilized for 10 min with 0.3% TritonX-100 in PBS. After blocking for 40 min with PBS+3% bovine serum albumin, cells were incubated with primary antibodies diluted in PBS+3% bovine serum albumin at 4 °C overnight in wet chamber. Cells were rinsed in 0.05% Tween20 in PBS and then permeabilized for 10 min with 0.3% TritonX-100 in PBS. Cells were incubated with secondary antibodies diluted in PBS+3% bovine serum albumin at room temperature in the dark for 1 h. For nuclear staining, cells were incubated with 4′,6-diamidino-2-phenylindole for 5 min and then washed with 0.05% Tween20 in PBS and again permeabilized with 0.3% TritonX-100 in PBS. After rinse in H_2_O, slides were mounted with ProLong Gold (Invitrogen) and imaged using a confocal microscope (LSM780, Carl Zeiss, Jena, Germany). For foci quantification, cells were fixed in 96 well plates (BD Falcon, Corning, NY, USA) using the same protocol, and images were taken with an Operetta (PerkinElmer, Waltham, MA, USA) or Image Xpress (Molecular Devices, Sunnyvale, CA, USA) high throughput microscope and analyzed using Columbus software (PerkinElmer) or Cell Profiler. More than 400 cells were counted at the Operetta, while more than 3000 cells were counted at the Image Xpress for each condition. To determine number of S-phase cells, U2OS cells were incubated with 10 μM EdU for 20 min, 24 h or 48 h, fixed and stained according to the manufactures' protocol (Invitrogen). The error bars represent s.e.m. from two-three independent experiments.

### Homologous recombination assay

Measurement of the frequency of HR-mediated DSB repair was performed as previously described.^36^ In brief, DR-GFP U2OS cells with an integrated HR reporter, DR-GFP, were transfected with indicated siRNAs. Forty-eight hours later, cells were transfected with I-SceI expression vector. After another 48 h, cells were harvested, fixed with 4% paraformaldehyde and subjected to fluorescence-activated cell sorting analysis to determine the efficiency of HR-mediated gene conversion (HR efficiency) induced by I-SceI digestion, which reconstitutes a functional GFP gene. GFP signal was quantitated with fluorescence-activated cell sorting Navios flow cytometer (Beckman Coulter, Inc., Indianapolis IN, USA) and analyzed with Kaluza software (Beckman Coulter, Inc.). For each experiment, 20 000 cells were analyzed per treatment and the frequency of recombination events was calculated from the number of GFP-positive cells divided by the number of cells analyzed. ISO and SSA treatment was started 24 h after siRNA transfection.

### Immunoblotting

Cells were collected and total cell extracts were obtained using radioimmunoprecipitation assay (RIPA) buffer (50 mM Tris-Hcl pH8.0, 150 mM NaCl, 0.5 w/v% sodium deoxycholate, 0.1 w/v% sodium dodecyl sulfate, 1.0 w/v% NP-40) with complete, ethylenediaminetetraacetic acid (EDTA) free (Roche, Basel, Switzerland) and Halt Phosphatase inhibitor cocktail (Thermo Scientific, Waltham, MA, USA). Laemmli Sample buffer (BIO-RAD, Sundbyberg, Sweden) was added and proteins were resolved by sodium dodecyl sulfate (SDS) gel electrophoresis, transferred onto Amersham Hybond-P (GE Healthcare) and probed using the appropriate primary and secondary antibodies coupled to either horse-radish peroxidase or fluorescent molecules. Signals were detected by SuperSignal West Pico Chemiluminescent Substrate (Thermo Scientific) or scanning of fluorescent signal using an Odyssey machine (Li-Cor).

### RNA extraction and quantitative real-time-PCR

Total RNA was isolated from cells using Direct-zol RNA Mini Prep (ZYMO RESEARCH, Irvine, CA, USA) according to the manufacturers' instructions. complementary DNA was generated using QuantiTect Reverse Transcription kit (Qiagen) and used as a template in real-time quantitative PCR analysis. The PCR reactions were prepared using SYBR Green (Invitrogen). GAPDH, hypoxanthine-guanine phosphoribosyltransferase (HPRT) or β-Actin were used as control genes for normalization. Real-time quantitative PCR reactions were performed on Rotoe-Gene Q (QiAGEN). Relative gene expression was calculated by using the ΔΔCt method. Data were normailized to GAPDH.

### Comet assay

U2OS cells were seeded in six-well plates (100 000 cells per well). Twenty-four hours later, cells were transfected with RNAseH1 and empty vector using jetPEI (Polyplus Transfect). Another 24 h later, indicated siRNA (non-targeting siRNA or SNRPA1 pool) was transfected using INTERFERin (Polyplus Transfect) and incubated for 48 h before harvesting. For splicing inhibitor SSA experiment, cells were treated with 100 nM SSA for 48 h before harvesting. After washing with 1 × PBS, cells were re-suspended in 1 × PBS at a concentration of ~1 × 10^6^ cells/ml. In total, 50 μl cell suspension was mixed with 250 μl 1.2% low-melting agarose at 37 °C. The mixture was added to pre-warmed (37 °C) agarose coated fully frosted slide (Thermo-Fisher Scientific, Waltham, MA, USA) and a coverslip was added on top of the mixture. Slides were kept on ice for 10 min before removing the coverslip and incubated in lysis buffer (10 mM Tris pH 10.0, 2.5 M NaCl, 0.1 M ethylenediaminetetraacetic acid, 10% dimethyl sulfoxide and 1% TritonX-100) at 4 °C overnight in the dark. Slides were then transferred in electrophoresis buffer (200 mM ethylenediaminetetraacetic acid, pH 10) and denatured for 30 min. Electrophoresis was run at 300 mA, 25 V for 30 min in electrophoresis buffer using a Comet Assay tank (Carl Zeiss). Slides were washed in neutralization buffer (0.4 M Tris-HCl pH 7.5) and counterstained with SYBR Gold (diluted 1:1000 in PBS) (Invitrogen). Images were acquired using a confocal microscope LSM780 (Carl Zeiss) and were quantified using CometScore software. At least 200 comets per sample were analyzed. Tail moment is calculated as per cent DNA in the tail multiplied by the tail length.

### Laser microirradiation and live-cell imaging

Live-cell imaging, microirradiation and determination of recruitment kinetics were carried out as previously described^37^ with a Zeiss LSM780 confocal laser scanning microscope, equipped with a UV-transmitting Plan-Apochromat 40 × /1.30 Oil DIC M27 objective.
