# Supplementary material for: The spliceosome U2 snRNP factors promote genome stability through distinct mechanisms; transcription of repair factors and R-loop processing
Source: Oncogenesis. 2016 Dec 19;5(12):e280–. doi: 10.1038/oncsis.2016.70 (PMC5177769; doi:10.1038/oncsis.2016.70)
Supplement: Supplementary Material [file oncsis201670x1.pdf]

Figure S1.

a

Gene	mean # RAD51 Foci	DR-GFP	complex
SF3B1	0.49	0.07	U2
SF3B3	0.16	0.24	U2
SF3B4	0.11	0.08	U2
SF3B5	0.39	0.38	U2
SF3B14	2.15	0.20	U2
SNRPA1	0.50	0.28	U2
SNRPB	0.91	0.19	U2
RBMX	0.74	0.06	ND
ISY1	0.34	0.20	pre-mRNA
CDC40	0.29	0.04	pre-mRNA
CRNKL1	0.84	0.08	pre-mRNA
SLU7	1.20	0.12	pre-mRNA
SUPT5H	1.42	0.14	elongation
SUPT6H	0.84	0.13	elongation
control	5.00	1.00	-

b

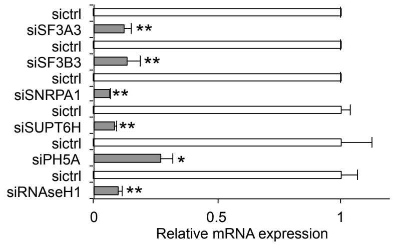

c

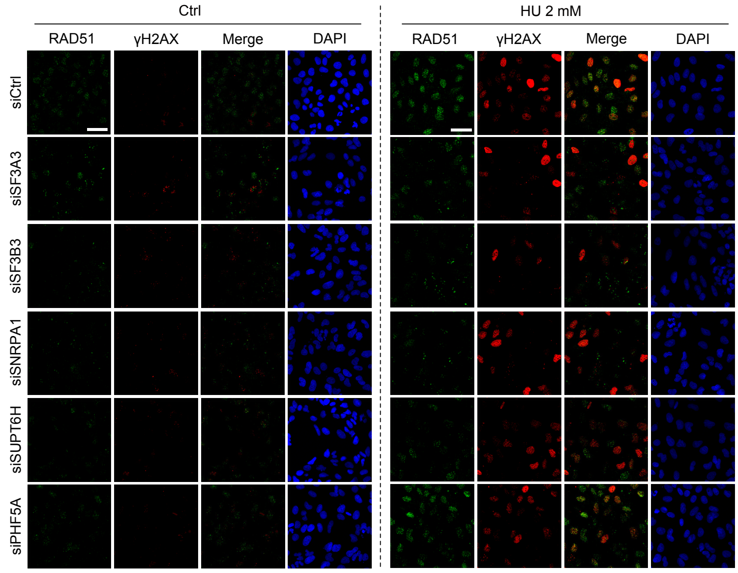

Figure S1. Splicing factors are required for RAD51 foci formation after replication stress. (a) List of all splicing factors amongst the top 100 in both screens. The genes highlighted in red were analyzed in this study. (b) Confirmation of siRNA mediated depletion of splicing factors and RNAseH1 with qRT-PCR compared to non-targeting siRNA. Relative mRNA levels to

GAPDH are shown. (c) U2OS cells were transfected with indicated siRNA and treated with 2 mM HU for 24 h. Cells were fixed and immunostained with RAD51 and γH2AX antibodies. The error bars represent SDs from three independent experiments (n=3). Statistically significant differences were determined using Student's t-test, *P<0.05, **P<0.01. Scale bar is 50 μm.

Figure S2.

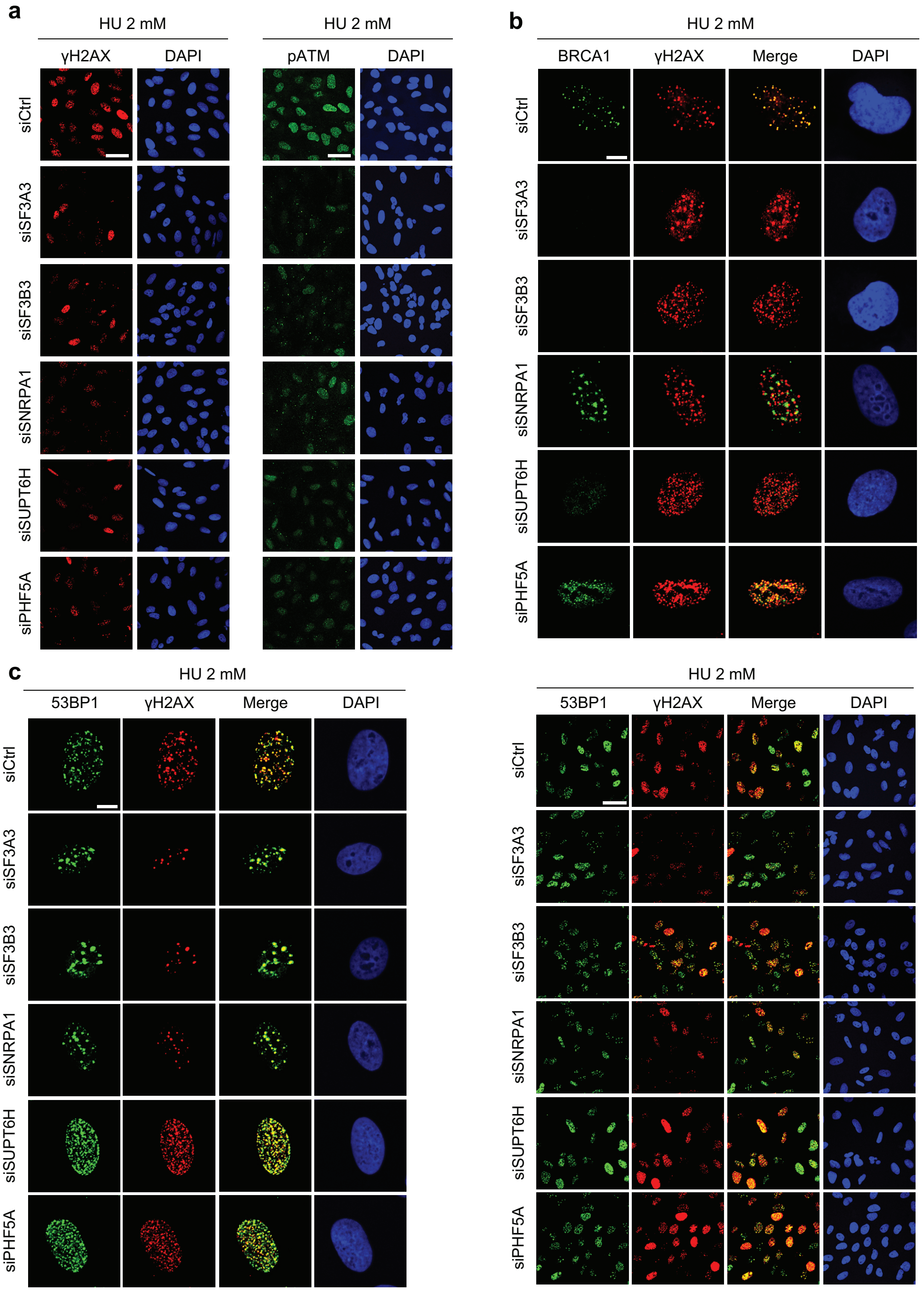

Figure S2. HU-induced γH2AX, pATM and BRCA1 foci formation is reduced, while 53BP1 is not significantly impaired in splicing factor depleted cells. U2OS cells were transfected with indicated siRNA and treated with 2 mM HU for 24 h. Cells were fixed and immunostained with (a) γH2AX, pATM, (b) BRCA1 and (c) 531BP1 antibodies. Scale bar is 10 μm and 50 μm, respectively.

Figure S3.

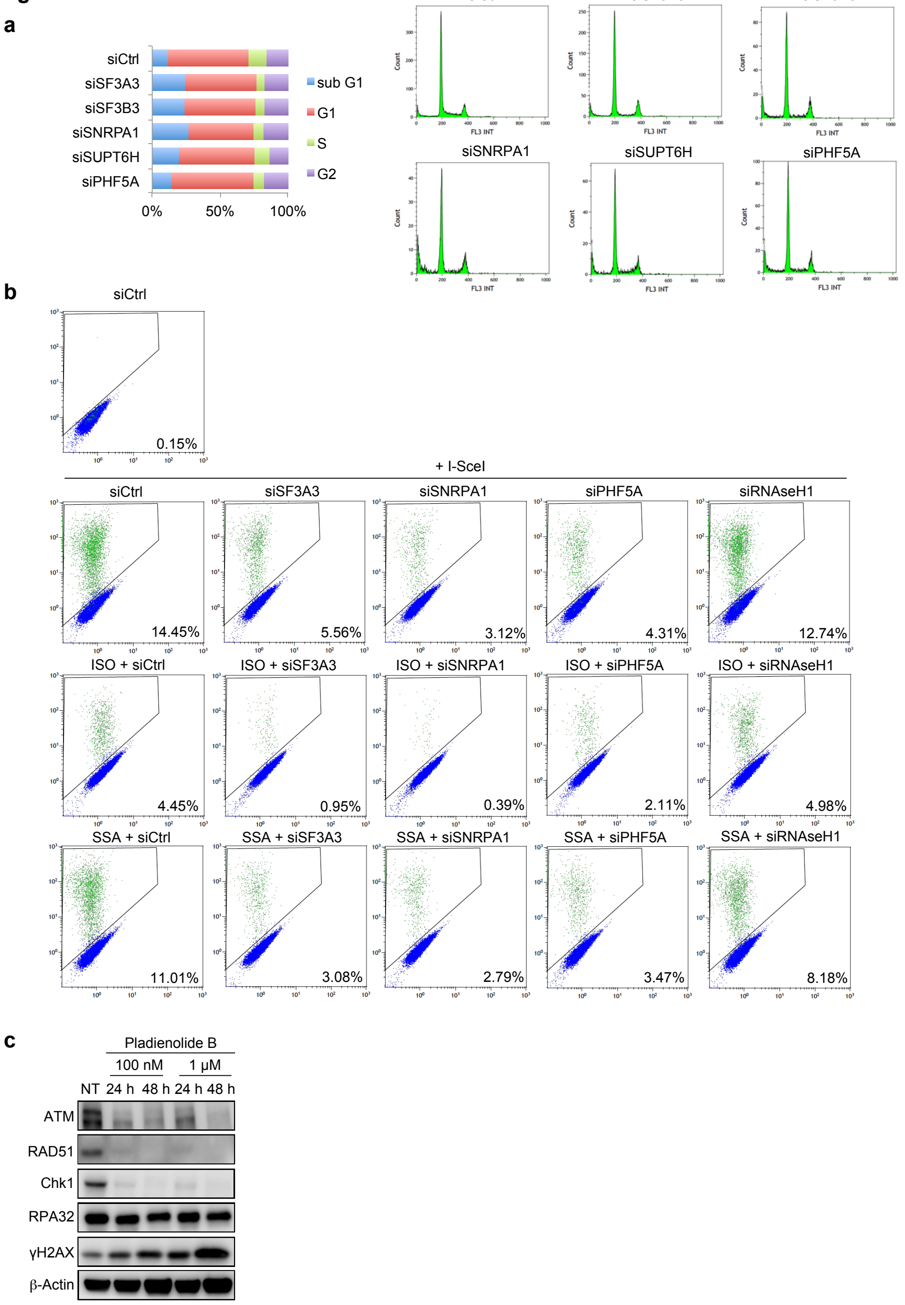

Figure S3. Effect of splicing factor depletion on cell cycle and different splicing inhibitors on HR and transcription. (a) U2OS cells were transfected with indicated siRNAs and 72 h later collected for FACS analysis. Representative FACS profiles and quantification of different cell cycle stagesare shown. (b) DR-GFP U2OS cells were treated with the indicated

siRNAs, 48 h later transfected with I-SceI vector and after another 48 h fixed and analysed using FACS. Representative FACS profiles are shown. (c) U2OS cells were treated with Pladienolide B (100 nM or 1 μM) for 24 to 48 h and probed for expression of ATM, RAD51, Chk1, RPA32, γH2AX and Actin.

Figure S4.

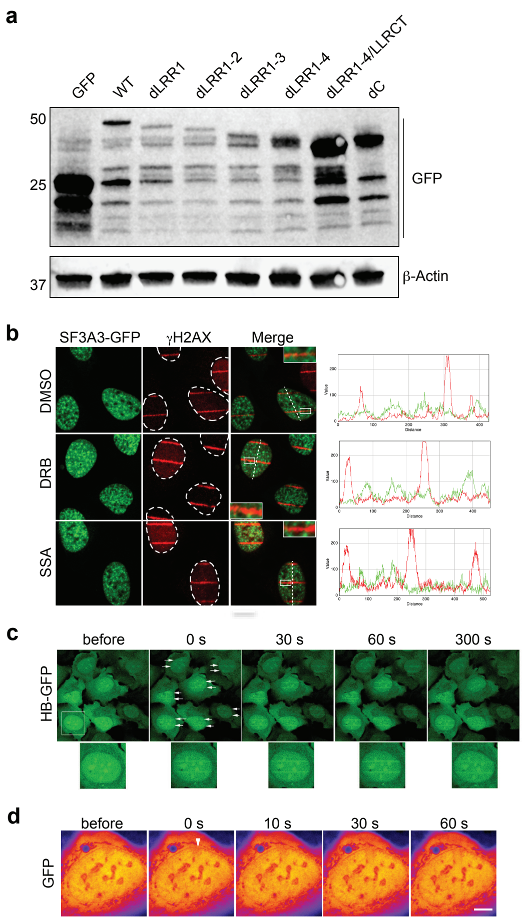

Figure S4. Accumulation and dissociation of SNRPA1 and SF3A3 at laser tracks and generation of R-loops at DNA damage sites. (a) Correct expression of GFP-tagged SNRPA1 deletion constructs. (b) U2OS cells expressing SF3A3-GFP were treated with DMSO, DRB (50 μM) or SSA (100 nM) for at least 1 h prior microirradiation. Cells were fixed and probed with antibodies againt γH2AX. Confocal images and line scans are shown. Inset in the merged image shows magnification of SF3A3-GFP and γH2AX signal at

microirradiated sites. (c) U2OS cells stably expressing HB-GFP were microirradiated and recruitment followed over time. (d) GFP alone is not recruited to laser-induced DNA damage sites in U2OS cells. Representative confocal images are shown. Scale bar 5 μM.

Figure S5.

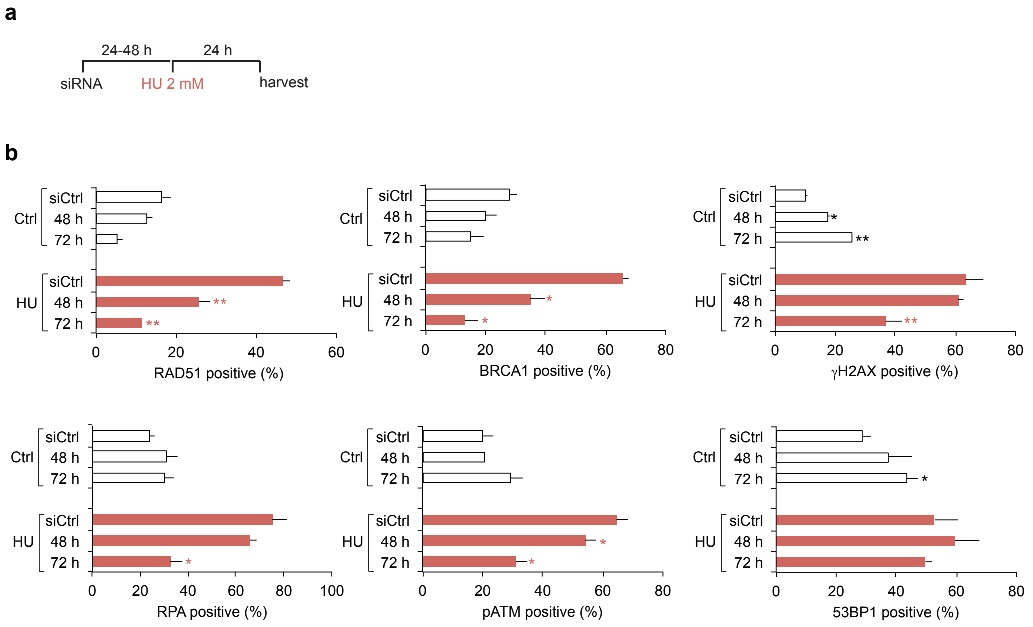

Figure S5. Accumulation of HR repair factors, RAD51, BRCA1, γH2AX, RPA and pATM at HU-induced damage sites is impaired in SNRPA1 depleted cells, while 53BP1 is not affected. (a) Experimental outline. (b) U2OS cells were transfected with siRNA for SNRPA1 for the indicated time periods before addition of 2 mM HU for 24 h. Cells were fixed and immunostained. Quantification of RAD51 (>12 foci), BRCA1 (>12 foci), γH2AX (>12 foci), pATM (>8 foci), RPA (>12 foci) and 53BP1 (>12 foci) positive cells. For each condition, more than 400 cells were analyzed. In splicing factor depleted cells replication stress, induced DNA repair is impaired 48 h after siRNA transfection due to downregulated transcription.
